# The Alexipharmic Mechanisms of Five Licorice Ingredients Involved in CYP450 and Nrf2 Pathways in Paraquat-Induced Mice Acute Lung Injury

**DOI:** 10.1155/2019/7283104

**Published:** 2019-04-28

**Authors:** Zi-Jing Liu, Jing Zhong, Mei Zhang, Ze-Hui Chen, Ji-Ye Wang, Han-Ying Chen, Xiao-Qin Wang, Bo Zhang

**Affiliations:** ^1^Key Laboratory of Xinjiang Endemic Phytomedicine Resources, Ministry of Education, School of Pharmacy, Shihezi University, Shihezi 832002, China; ^2^Pharmacology Department, School of Pharmacy, Shihezi University, Shihezi 832002, China

## Abstract

Oxidative stress is an important mechanism in acute lung injury (ALI) induced by paraquat (PQ), one of the most widely used herbicides in developing countries. In clinical prophylaxis and treatment, licorice is a widely used herbal medicine in China due to its strong alexipharmic characteristics. However, the corresponding biochemical mechanism of antioxidation and detoxification enzymes induced by licorice's ingredients is still not fully demonstrated. In this study, the detoxification effect of licorice was evaluated *in vivo* and *in vitro*. The detoxification and antioxidation effect of its active ingredients involved in the treatment was screened systematically according to Absorption, Distribution, Metabolism, and Excretion (ADME): predictions and evidence-based literature mining methods *in silico* approach. Data shows that licorice alleviate pulmonary edema and fibrosis, decrease Malondialdehyde (MDA) contents and increase Superoxide Dismutase (SOD) activity in PQ-induced ALI mice, protect the morphologic appearance of lung tissues, induce cytochrome 3A4 (CYA3A4) and Nuclear factor erythroid 2-related factor 2 (Nrf2) expression to active detoxification pathways, reduce the accumulation of PQ *in vivo*, protect or improve the liver and renal function of mice, and increase the survival rate. The 104 genes of PPI network contained all targets of licorice ingredients and PQ, which displayed the two redox regulatory enzymatic group modules cytochrome P450 (CYP450) and Nrf2 via a score-related graphic theoretic clustering algorithm *in silico*. According to ADME properties, glycyrol, isolicoflavonol, licochalcone A, 18beta-glycyrrhetinic acid, and licoisoflavone A were employed due to their oral bioavailability (OB) ≥ 30%, drug-likeness (DL) ≥ 0.1, and being highly associated with CYP450 and Nrf2 pathways, as potential activators to halt PQ-induced cells death *in vitro*. Both 3A4 inhibitor and silenced Nrf2 gene decreased the alexipharmic effects of those ingredients significantly. All these disclosed the detoxification and antioxidation effects of licorice on acute lung injury induced by PQ, and glycyrol, isolicoflavonol, licochalcone A, 18beta-glycyrrhetinic acid, and licoisoflavone A upregulated CYP450 and Nrf2 pathways underlying the alexipharmic mechanisms of licorice.

## 1. Introduction

Paraquat (N,N-dimethyl-4,4′-bipyridinium dichloride; PQ) is one of the most widely used herbicides in developing countries. However, it is also a highly toxic compound caused by pulmonary impairment, leading to irreversible pulmonary fibrosis and characteristically occurred between several days and 3 weeks after administration or ingestion, which accounts for 300,000 deaths occurring in the Asia-Pacific region alone each year [1]. The diagnosis of PQ poisoning mainly is according to the PQ concentration in the blood, lung X-ray, and dithionite urine test [2]. With the development of science, a GC-MS-based metabolomic approach was applied to assess the metabolite changes in human plasma following PQ poisoning [3]. According to the literature, SVM discrimination model performed with high classification accuracy can be applied to distinguish the PQ poisoned patients from a healthy person [3]. Also a potential use of ELM methods for PQ poisoning detection in biomedical applications, which possess high classification accuracy, AUC, sensitivity, and specificity, can be applied in clinical practice to distinguish the PQ-poisoned patients from healthy persons [4]. But many of them require a long time, money, or staff. At present, a simple but reliable highly sensitive fluorescence-enhancing method has been developed for the detection of PQ concentration by using host-guest chemistry with cucurbit[8]uril [2]. Management strategies for PQ poisoning include extracorporeal elimination, immunosuppressants, antioxidants, and hemoperfusion (HP) within 2-4 h after intoxication [6–8]. However, there is no sufficient evidence indicating good clinical outcomes, and mortality rates remain very high despite current treatment methods [5, 9]. Thus, it is important to find an antidote and identify a better therapeutic mechanism of PQ poisoning.

Considering the strong detoxification of the liver, the induction of drug-metabolizing enzymes and efflux transporters promotes the detoxification and excretion of toxic substances, which is considered to be one of the important pathways in drug detoxification [10]. Typical phase I metabolic enzymes are cytochrome P450 (phase I metabolic enzymes), ALDH, and so on. In P450, the actual effects of the accumulation of toxic metabolic products of drugs and the detoxification process depend on the balance between phase I and phase II enzymes. In the literature, acute paraquat poisoning may induce the activities of CYP2C19 and inhibit the activities of CYP2B6, CYP2C9, CYP2D6, and CYP3A4 in rats [11]. This may provide a reasonable suggestion for the development of new drugs for acute paraquat poisoning.

Oxidative stress is also thought to participate in the mechanism of PQ-induced ALI [5]. PQ can enter cells and participate in a series of redox reactions. In this process, PQ consumes nicotinamide adenine dinucleotide phosphate and cytochrome P450 reductase, causing oxidative stress reaction. MDA level can indirectly reflect the degree of peroxidation, and elevated MDA level also causes cell metabolism disorders. SOD is the main endogenous antioxidant which can effectively remove O_2_^·-^ and reduce oxidation reactions, causing the subsequent acute inflammatory response. When these antioxidants are imbalanced, there will be airway inflammation, airway hyperresponsiveness and tissue damage, neutrophil infiltration, and ultimately the formation of lung injury [12]. It has been found that Nrf2, an important transcription factor of oxidative stress, is known to be essential for the antioxidant responsive element-mediated induction of oxidative stress enzyme genes and plays an important role in the development of PQ-induced ALI. Many studies showed their results by inhibiting oxidative stress to slow down the process of ALI [13].

Detoxification is an important mechanism involved in polypharmacology [14]. And it may be beneficial for detoxification by reducing oxidative stress, minimizing toxicity by regulating the expression CYP450 mRNAs [15]. Licorice is one of the most widely used herbal medicines in China due to its strong alexipharmic characteristics. It appeared in “Shang Han Za Bing Lun” and reduces toxicity of other drugs in prescriptions. There are many active components in licorice, but the mechanism of detoxification is unknown. At present, many components have been proved to exhibit detoxification activity; triterpenoids and flavonoids in the licorice are responsible for combining with toxic substances as their alexipharmic mechanism [16]. Glycyrrhizin obviously induces CYP3A activity *in vivo* [17]. 18*β*-Glycyrrhizinate is responsible for the concentration-dependent induction of CYP3A [18]. However, *in vivo* data disclosed medicagenin (II) and munchiwarin (III) showing low oral bioavailability; other active molecules should be searched to take the alexipharmic role in licorice [19].

Nrf2 is a basic leucine zipper transcription factor and plays a key role in cellular defenses against electrophile induced-oxidative stress [20]. According to the literature, the active ingredients in licorice, such as isoliquiritigenin, stimulate detoxification system via Nrf2 activation, which could be a potential protective mechanism of licorice [21]. A number of compounds such as licochalcone A [22], licochalcone B, glycyrrhetinic acid, glycyrrhizin, and isoliquiritigenin significantly activate Nrf2 [23], induce Nrf2 expression [21], and were potent Nrf2 activators. Furthermore, drug-metabolizing enzymes are essential for most of the biotransformation steps involving xenobiotics and endogenous molecules [24]. Recent studies have found that Nrf2 regulates the expressions of a variety of drug-metabolizing enzymes (phase II metabolic enzymes) and transporters *in vivo* and also the primary factor inducing the cell survival system under Glutathione (GSH) depletion [21, 25]. At present, hundreds of active ingredients in licorice have been isolated and identified. Many of those molecules have relatively fast metabolism and low bioavailability in the liver, although their content is high, the final duration is short. According to ADME, there are still many compounds including 18beta-glycyrrhetinic acid, glycyrol, licoisoflavone A, and isolicoflavonol with high oral bioavailability values in licorice. But there is hardly any research on the potential detoxification value of these molecules. Molecules with good ADME properties are the important standard for drugs. It is urgent to find the active ingredients with relative higher OB and higher activities targeting both CYP450 and Nrf2/ARE metabolic pathways in the treatment of PQ poisoning [24].

Systems biology, such as network pharmacology, promotes an understanding of the function and behavior of a biological system [26], so did the evidence-based analytic platforms and network science algorithm models [27, 28]. Those technological platforms provide holistic approaches to study the essence of herb medicine and the functions of natural products *in silico* [26]. The liver is where drugs are metabolized; it is the main active place of drug-metabolizing enzymes, and HepG2-derived hepatocellular carcinoma is a hepatoblastoma suitable for the study of hepatocyte metabolism and detoxification. A549 cells are widely used in the model of type II pulmonary epithelial cells *in vitro* and as transfection hosts. The main target organ of PQ poisoning is the lung, and A549 cells are adenocarcinoma and human alveolar basal epithelial cells. And type II alveolar epithelial cells are specific targets of paraquat toxicity [29]. So, A549 cells and HepG2 cells were chosen to explore the detoxification properties of licorice *in vitro*. In the present study, we aim to evaluate the detoxification and antioxidation effects of licorice *in vivo* and characterize the active ingredients and their pharmacological actions *in silico* and *in vitro* underlying the mechanism of licorice detoxification.

## 2. Methods and Materials

### 2.1. Materials

Licorice extract (Cat#L9030) is a water-soluble powder, extracted from the dried roots and stems of *G. uralensis Fisch.*, *G. inflate Bat.*, and *G. glabra L.* (*Glycyrrhiza Linn.*, *Leguminosae*) by water; glycyrol (GLL, purity ≥ 99%, PubChem CID: 5320083), isolicoflavonol (ILF, purity ≥ 98%, PubChem CID: 5318585), licochalcone A (LCA, purity ≥ 99%, PubChem CID: 5318998), licoisoflavone A (LIA, purity ≥ 98%, PubChem CID: 5281789), 18beta-glycyrrhetinic acid (18*β*-GA, purity ≥ 99%, PubChem CID: 10114), and MTT [3-(4.5-dimethylthiazol-2-yl)-2.5-diphenyl tetrazolium bromide] and Modified Masson's Trichrome Stain Kit were purchased from Solarbio Co. Ltd. (Beijing, China). The cDNA amplification kit, primers, and RNA extraction kit were purchased from Shanghai Sangon Co. Ltd. (Shanghai, China). Dulbecco's Modified Eagle's Medium Nutrient Mixture F-12 (DMEM/F12) and Minimum Essential Medium (MEM) were purchased from Gibco BRL (Grand Island, NY, USA). All of the primary antibodies CYP3A4, Nrf2, glyceraldehyde-3-phosphate dehydrogenase (GAPDH), anti-mouse, and anti-rabbit secondary antibodies were purchased from Cell Signaling Technology Inc. (Danvers, MA, USA). Ketoconazole (KZ, purity ≥ 98%, PubChem CID: 3823) was purchased from Shanghai Yuanye Biological Industries Co. Ltd. (Shanghai, China). The human type II alveolar adenocarcinomic basal epithelial cells (A549) and Human hepatocellular liver carcinoma cell (HepG2) were purchased from the Cancer Cell Repository (Shanghai Cell Bank, Shanghai, China). Nrf2 knockdown A549 cells were transfected with Nrf2shRNA [30]. Paraquat (PQ, Sigma-Aldrich Company) (PubChem CID: 15938) and methylene blue (MB) were purchased from Sigma-Aldrich. The MDA kit, SOD kit, AST/GOT kit, ALT/GPT kit, CRE kit, and BUN kit were purchased from Nanjing Jiancheng Bioengineering Institute (Jiangsu, China). Dexamethasone Sodium Phosphate Injection was purchased from Tianjin King York Group Co. Ltd. Cucurbit[8]uril was obtained by Prof. Shiguo Sun.

### 2.2. Collection and Library Construction of Active Ingredients of Licorice

The list of licorice chemicals used in the treatment of respiratory diseases was collected from the literature, the Traditional Chinese Medicine Systems Pharmacology Database and Analysis Platform (TCMSP, accessed on May 1, 2015, http://lsp.nwu.edu.cn/tcmsp.php), and the Chinese Academy of Sciences' Chemistry Database (accessed on May 1, 2015, http://www.organchem.csdb.cn). The chemical structures were obtained from the Chemical Book (http://www.chemicalbook.com).

### 2.3. Network Construction of Protein Interaction in Licorice Ingredients and Paraquat

The ingredients of OB ≥ 30% and DL ≥ 0.1 in licorice through TCMSP database (Supplementary Table 1) and PQ were submitted to the Agilent Literature Search software v.3.1.1 (http://www.agilent.com/labs/research/litsearch.html), which is a powerful automatic metasearch tool for querying multiple text-based PubMed and USPTO, for associations among genes of interest and constructing a network. A graph theoretic clustering algorithm, MCODE (Molecular Complex Detection), was used to analyze the characteristics of the networks. The equation *i* = 2*n*/(*ki*(*ki* − 1)) was used, where *ki* is the vertex size of the neighborhood of vertex *i* and *n* is the number of edges in the neighborhood [28]. Interactomes with a module score ≥ 3.3 and at least two nodes were selected as significant predictions [27]. STRING (accessed on May 1, 2015, http://string-db.org/) was used to analyze the results of oxidoreductase.

### 2.4. ADME Property Prediction of Licorice Ingredients

To identify the detoxifying active ingredients of licorice, we used the TCMSP database to predict OB *in vivo* (%F) and DL of the ingredients. Using this database, we chose those with OB ≥ 30% and DL ≥ 0.1 as the candidate ingredients, thus reducing the number of original chemicals from the licorice to a smaller subset significantly [31].

### 2.5. Evidence-Based Finding of Potential Molecules by QSAR and Molecular Docking Study

The quantitative structure-activity relationship (QSAR) model was used as a tool to screen for ingredients depleted of GSH. Organization for Economic Cooperation and Development QSAR (v. 3.2.0, 2013, http://www.qsartoolbox.org/) was used to quantitatively research the interactions of small organic molecular substance with biomacromolecules. The module of protein-binding potency was used in the current study [32].

Molecular docking was used as a tool to screen for ingredients that have strong energy binding to CYP3A4, which is a member of the cytochrome P450 family of oxidizing enzymes. Several other members of this family are also involved in drug metabolism, but CYP3A4 is the most common and the most versatile one [33]. Ingredients in licorice, together with CYP3A4 to carry out virtual molecular docking, use carbamazepine as a positive control. Docking and scoring of the ingredients in licorice were performed using Surflex-Dock in SYBYL -X 2.0. The models of the ingredients were then docked into the crystal structure protein model with Surflex-Dock (default settings with reign flexibility sampled), and the resulting 10 best poses were sorted by the Surflex-Dock scoring function in -log_10_(*K*_d_) units to simulate binging affinities [34]. Four ingredients were selected according to these filtering criteria.

### 2.6. Cell Lines, Culture, and Treatment

A549 and HepG2 cells were maintained in DMEM/F12 and MEM, respectively, containing 10% fetal bovine serum, 100 U/mL penicillin, and 100 U/mL streptomycin at 37°C and 5% CO_2_ in a suitable incubator. A549 cells and Nrf2 knockdown A549 cells were seeded in 96-well cell plates (5 × 10^4^/well), respectively, and grown for 12 h. PQ (0-40 *μ*M) was mixed with the cells and incubated for 24 h. KZ was the CYP3A4 inhibitor. HepG2 cells were seeded in 96-well cell plates (5 × 10^4^/well) and grown for 12 h. After that they were pretreated with 10 *μ*M KZ for 4 h and exposed to 0-40 *μ*M PQ for 24 h. Cell proliferation was determined by MTT assay.

A549 cells and HepG2 cells were seeded in 96-well cell plates, respectively (5 × 10^4^/well), and grown for 12 h, after they were pretreated with 10 *μ*M GLL, ILF, LCA, LIA, and 18*β*-GA for 4 h and exposed to 0-40 *μ*M PQ for 24 h. Cell proliferation was determined by MTT assay.

A549 and HepG2 cells were seeded in 96-well cell plates (5 × 10^4^/well) and grown for 12 h, after which 0–100 *μ*M GLL, ILF, LCA, LIA, and 18*β*-GA were separately mixed with the cells and incubated for 24 h. Cell proliferation was determined by MTT assay.

Survival fraction = mean OD in text wells - mean OD in free cell wells/mean OD in control wells - mean OD in free wells [35].

### 2.7. Reverse Transcription Polymerase Chain Reaction and Western Blot Analysis

A549 cells were plated in six-well tissue culture plates. 5 *μ*M and 10 *μ*M GLL, ILF, LCA, and LIA and 50 *μ*M and 100 *μ*M 18*β*-GA were separately incubated with the cells for 8 h. The levels of Nrf2, glutathione reductase (GR), glutathione peroxidase (GPX), and NAD (P)H:quinone oxidoreductase (NQO1) mRNA were analyzed by Real-Time PCR (RT-PCR).

Nrf2 knockdown A549 cells were plated in six-well tissue culture plates, after which 10 *μ*M GLL, ILF, LCA, and LIA and 50 *μ*M 18*β*-GA were separately incubated with the cells for 8.0 h. The levels of glutamate cysteine ligase modifier (GCLM) and NQO1 mRNA were analyzed using RT-PCR.

HepG2 cells were plated in six-well tissue culture plates and grown for 12 h, after which 10 *μ*M GLL, ILF, LCA, and LIA and 50 *μ*M 18*β*-GA were separately incubated with the cells for 24 h. The levels of CYP3A4 mRNA were analyzed by RT-PCR [36].

Quantitative real-time RT-PCR was performed using a single-tube SYBR Green kit (QIAGEN, USA), Rotor Gene Q Real-Time PCR system (Rotor Gene Q, QIAGEN, USA), and specific primer sets (the same primers used in the conventional RT-PCR). Only experiments in which a distinct single peak was observed with a melting temperature different from that of the no-template control were analyzed. The relative amount of target mRNA was calculated by the 2^–△△Ct^ method. Each sample was analyzed using glyceraldehyde-3-phosphate dehydrogenase (GAPDH) as an endogenous reference gene for mRNA normalization.

HepG2 cell was treated with 10 *μ*M GLL, ILF, LCA, and LIA and 50 *μ*M 18*β*-GA separately for 24 h, after which the cells were harvested and lysed in RIPA buffer. The levels of CYP3A4 were analyzed by western blot with primary antibody (1 : 1000) and its corresponding secondary antibody (1 : 10000) according to manufacturer's instructions [36].

### 2.8. Animal Treatments

KM mice (6-8 weeks old) were purchased from Xinjiang Medical University (Xinjiang, China), weighing 18-25 g (animal qualified number: SCXK 2016-0003). The animals were randomly divided into 6 groups (*n* = 8) as follows: (1) control group, (2) PQ group, (3) dexamethasone group, (4) licorice extract (20 mg/kg), (5) licorice extract (40 mg/kg), and (6) licorice extract (60 mg/kg). The animals were housed in a ventilated room at 22 ± 2°C with a 12 h dark/12 h light cycle and provided with free access to fresh water and standard rodent chow. Mice in the control group received saline solution. In the PQ group, mice received PQ (45 mg/kg, i.g.). Licorice extract (20, 40, and 60 mg/kg) groups were treated with licorice extract (20, 40, and 60 mg/kg/d, i.g.) 1 h before mice received PQ. The dexamethasone (DXMS) group was treated with DXMS (0.5 mg/kg/d, ip) 1 h before mice received PQ. The PQ group, PQ+licorice extract group, and DXMS group were treated once with an intragastric infusion of PQ solution (40 mg/kg); licorice extract and DXMS were dissolved in saline (NaCl 0.9%).

The survival of the mice in each group was observed over eight observation periods: 0, 1, 2, 3, 4, 5, 6, and 7 days after treatment administration. Mice were anaesthetized after treatment for 7 days; lung tissues, liver tissues, and renal tissues were collected. And blood samples were then collected from the inferior vena cava from each group and stored at 4°C overnight for serum preparation and PQ concentration detection. Weighing the lung tissue of mice, use lung W/D = wet weight/dry weight to calculate the lung W/D in mice. Histological examination of the lung, liver, and renal tissues from each group was performed to evaluate tissue damage.

Parts of lung tissues were used for measuring oxidative stress biomarkers. Malondialdehyde (MDA) level and Superoxide Dismutase (SOD) activity were determined according to the thiobarbituric acid (TBA) method and xanthine oxidase method as manufacturer's instructions (Nanjing Jiancheng Bioengineering Institute, China). The levels of CYP3A4 and Nrf2 were analyzed by western blotting according to manufacturer's instructions [36]. Serum was used for measuring renal and liver function evaluation. Urea nitrogen (BUN), creatinine (CRE), aspartate aminotransferase (AST/GOT), and alanine aminotransferase (ALT/GPT) levels were determined according to the urease and sarcosine oxidase methods as manufacturer's instructions (Nanjing Jiancheng Bioengineering Institute, China).

According to the literature, for fluorescence detecting of paraquat using host-guest chemistry with cucurbit[8]uril, we used the methods in [2]; 2MB@CB[8] (200 *μ*L, 5 *μ*M) was added into 0.1 mL serum of mice to determine the concentration of PQ in serum. According to the literature, the fluorescence reaction of 2MB@CB[8] and PQ also occurs in live mice. After injecting 2MB@CB[8] solution into mice (0.1-0.2 mL), real-time fluorescence imaging of PQ *in vivo* was carried out to demonstrate the residual of PQ in each group of mice by using a NightOWL II LB 983 system equipped with a NC 100 CCD deep-cooled camera (Berthold Technologies, Bad Wildbad, Germany).

### 2.9. Statistical Analysis

Statistical analysis was conducted with SPSS version 18.0 software and GraphPad Prism 5, using one-way analysis of variance (ANOVA) for multiple group comparisons or Student's *t*-test for two-group comparisons. All data are expressed as the mean ± standard error [37]. *P* < 0.05 was considered statically significant.

## 3. Results

### 3.1. Survival Rate of Mice

**Figure 1:**
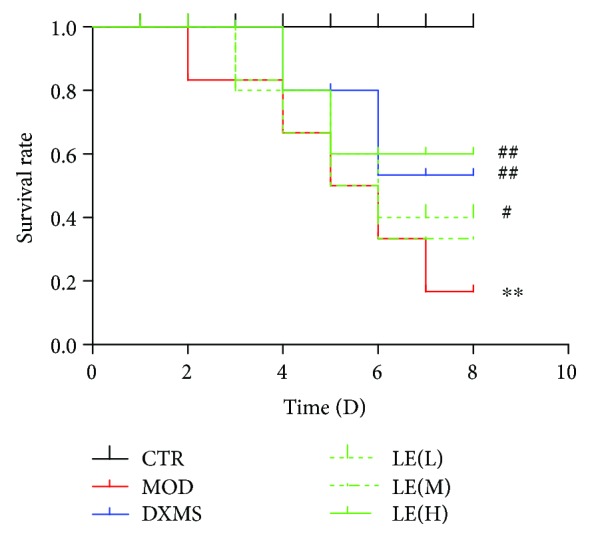
Survival rate. Effects of licorice on survival rate of PQ-induced ALI mice. Mice were given licorice extract (20, 40, and 60 mg/kg) 1 h prior to administration of PQ. Record the number of deaths after the mice were poisoned by PQ. The Kaplan-Meier program was utilized to compare the differences in survival rates between groups. The values presented are the means ± SEMs (*n* = 8 in each group). Compared with the CTR group, ^∗^ *P* < 0.05, ^∗∗^ *P* < 0.01; compared with the MOD group, ^#^ *P* < 0.05, ^##^ *P* < 0.01.

On the 7^th^ day, after PQ intoxication, licorice extract treatment had significantly increased the survival rate at day 8, with 60% survival in the PQ+licorice extract group (*P* < 0.01) compared with 20% in the PQ group. Licorice extract treatment improved the survival rate to 60% in the PQ+licorice extract group compared with 20% in the PQ group; the improvements were statistically significant (Figure 1). This result confirmed the protective effect of licorice on mortality in mice with PQ poisoning.

### 3.2. Licorice Attenuates the PQ-Induced Pulmonary Damage

**Figure 2:**
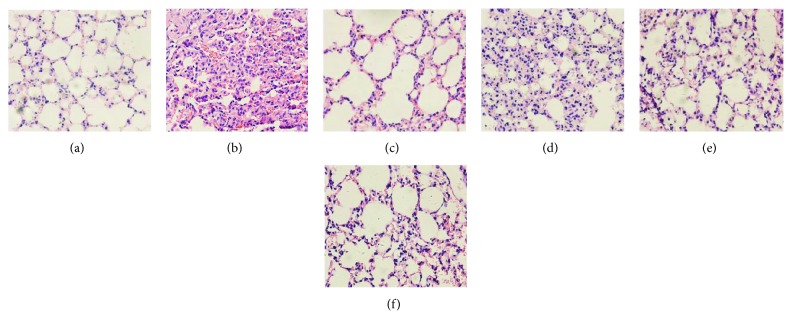
Lung histological examination from H&E staining. Histological appearance of the lung sections from the (a) normal group, (b) model group, (c) DXMS group, (d) licorice extract (20 mg/kg), (e) licorice extract (40 mg/kg), and (f) licorice extract (60 mg/kg). Widespread thickening of the alveolar septum and infiltration of inflammatory cells were observed in the model group. Treatment with licorice extract reduced the histological alterations induced by PQ. The lung sections were analyzed by hematoxylin and eosin (H&E) staining (magnification is ×400).

We used H&E staining to display pulmonary damages in each group of mice (Figure 2). In the control group, the alveoli showed clear boundaries and intact structures. Thickening of alveolar walls and infiltration of inflammatory cells were not observed nor were exudates from alveolar cavities. In the PQ group, incrassation was revealed in alveolar septum accompanied with discontinuous destruction. A large number of inflammatory cells such as neutrophils, macrophages, and lymphocytes infiltrated the alveolar cavities. Thickening alveolar walls, collapsed alveolar diffused pulmonary hemorrhage, and destruction of alveolar epithelial cells were observed significantly. In licorice extract-treated groups, compared with the PQ group, pulmonary damages were significantly reduced with administrated dosages from 20 to 60 mg/kg. The pathological thickening of the alveolar septum by PQ was alleviated. Inflammatory infiltration was significantly limited in the alveolar cavities. Few atrophied alveolar structures and thickened alveolar septum were observed.

### 3.3. Licorice Reduced PQ-Induced Pulmonary Edema of Mice

**Figure 3:**
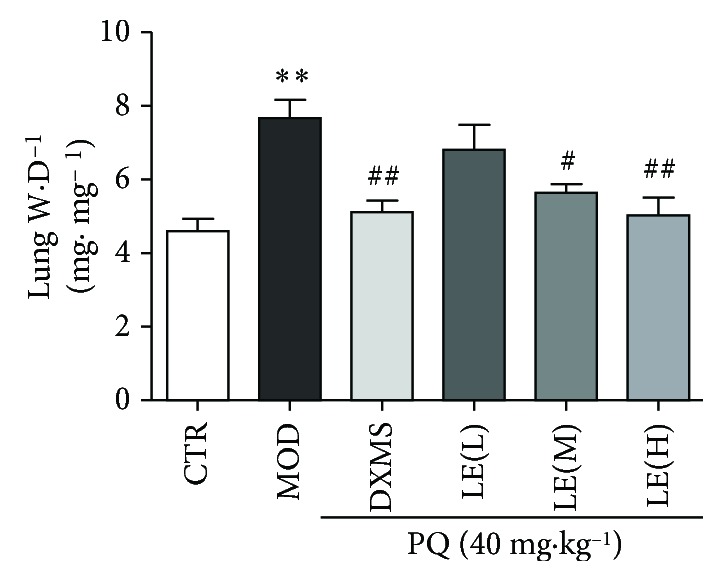
Licorice extract reduces lung W/D in mice. Effects of licorice on the lung W/D ratio of PQ-induced ALI mice. Mice were given licorice extract (20, 40, and 60 mg/kg) 1 h prior to administration of PQ. The lung W/D ratio was determined 7 days after the PQ challenge. The values presented are the means ± SEMs (*n* = 8 in each group). Compared with the CTR group, ^∗^ *P* < 0.05, ^∗∗^ *P* < 0.01; compared with the MOD group, ^#^ *P* < 0.05, ^##^ *P* < 0.01.

Lung wet/dry weight ratio is used to measure the edema degree of lung tissue in mice. Compared with the control, the lung wet/dry weight ratios were significantly increased after PQ gavage (*P* < 0.01). The increase of W/D was significantly reduced by licorice extract treatment at high doses (*P* < 0.01) (Figure 3). This result showed licorice extract reduce pulmonary edema in mice in different degrees.

### 3.4. Licorice Reduced PQ-Induced Oxidative Stress in the Lung Tissues of Mice

**Figure 4:**
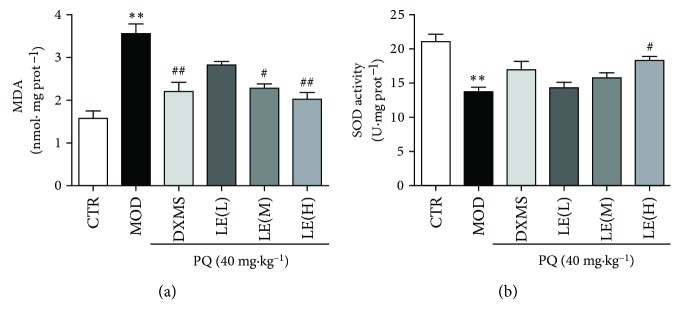
Licorice extract reduces the level of MDA and increases the activity of SOD. Effects of treatment with licorice extract on the levels of MDA (a) and SOD activity (b) of the lung tissue. Treatment with licorice extract reduced the level of MDA and increased the activity in the lung tissues of PQ-induced lung injury. Data are means ± SEM; *n* = 8. Compared with the CTR group, ^∗^ *P* < 0.05, ^∗∗^ *P* < 0.01; compared with the MOD group, ^#^ *P* < 0.05, ^##^ *P* < 0.01.

To evaluate the effect of licorice on PQ-induced oxidative stress damage, the level of MDA and SOD was measured which were biological indicators of oxidative stress. MDA level in the model group was significantly higher than that in the control group (*P* < 0.01). Compared with the PQ group, the MDA level in the licorice extract (20, 40, and 60 mg/kg) groups, especially in the licorice extract (60 mg/kg) group, was much lower (*P* < 0.01). According to Figure 4, the activity of SOD in the model group was significantly lower than that in the control group (*P* < 0.01). Compared with the PQ group, the licorice extract (60 mg/kg) group has increased SOD activity (*P* < 0.05) remarkably. As we can see, oxidative stress has been shown to be increased in the model group; licorice extract (60 mg/kg) decreased the level of MDA and increased the activity of SOD significantly.

### 3.5. Licorice Alleviated Pulmonary Fibrosis in Mice Induced by PQ

**Figure 5:**
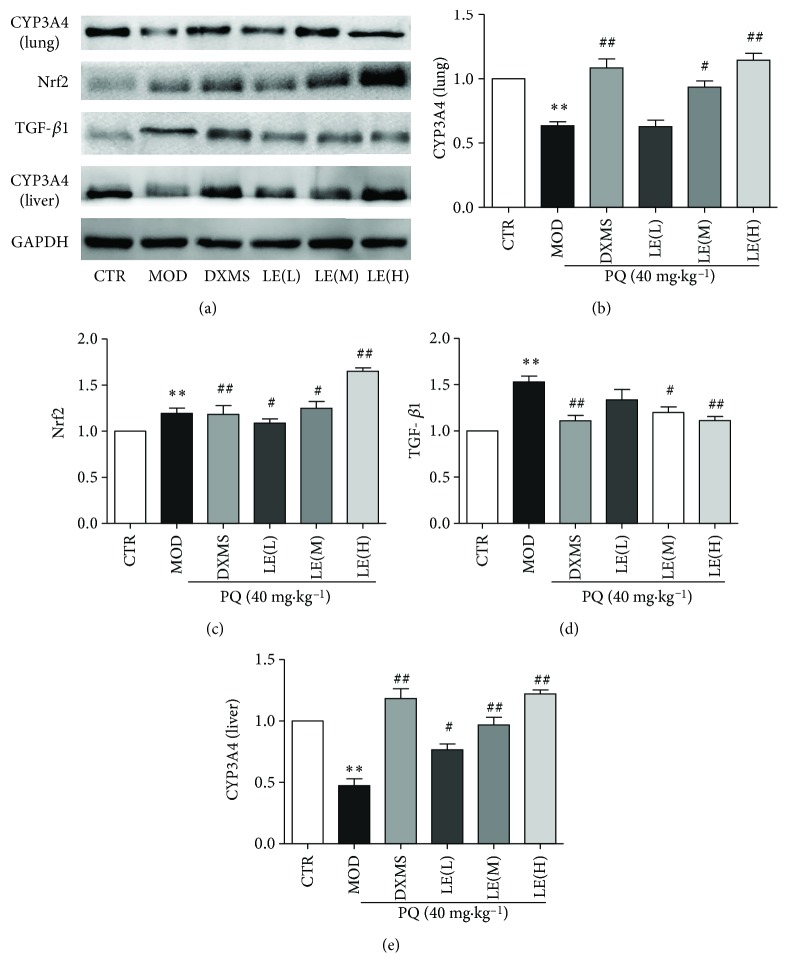
Licorice induce CYP3A4 and Nrf2 expression in mice and inhibit TGF-*β*1 expression. After processing the lung tissue of mice, Nrf2, CYP3A4, and TGF-*β*1 protein expression in the lung tissues and CYP3A4 protein expression in the liver tissues were measured by western blotting. CYP3A4 (lung): CYP3A4 expression in the lung tissues; CYP3A4 (liver): CYP3A4 expression in the liver tissues. Results show that licorice induce Nrf2 and CYP3A4 expression in a dose-dependent manner but inhibit TGF-*β*1 expression especially in licorice extract (60 mg/kg) (*P* < 0.01). Data points and error bars represent the mean ± S.E.M. of at least three experiments (*n* = 3) for each concentration tested; (^∗^ *P* < 0.05).

In order to evaluate pulmonary fibrosis degree in mice, western blotting was used to measure the level of TGF-*β*1, which is a potent profibrotic cytokine, in the foci containing these activated fibroblasts [38]. Compared with the control group, PQ significantly increased the level of TGF-*β*1 expression (*P* < 0.01); after the treatment of licorice extract (60 mg/kg), TGF-*β*1 expression was decreased remarkably (*P* < 0.01). It suggests that licorice reduces pulmonary fibrosis after PQ intake (Figure 5).

**Figure 6:**
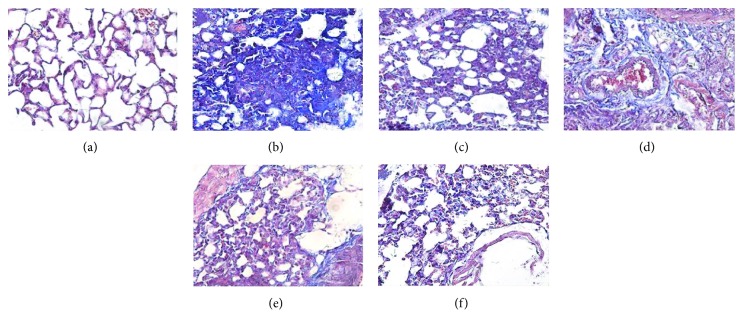
Photomicrographs of lung sections stained with Masson trichrome. The lung sections from the (a) normal group, (b) model group, (c) DXMS group, (d) licorice extract (20 mg/kg), (e) licorice extract (40 mg/kg), and (f) licorice extract (60 mg/kg) were stained with Masson trichrome. Fibrosis in the licorice extract group was decreased in a dose-dependent manner (magnification is ×400).

In order to show more directly that licorice alleviated pulmonary fibrosis after PQ intake, Masson trichrome staining was used to assess collagen deposition. Masson trichrome staining of formalin-fixed tissues revealed greater amounts of collagen deposition in the lung tissues of the model group (Figure 6). Fibrosis was decreased in a dose-dependent manner after the treatment of licorice extract.

### 3.6. Licorice Induced CYP3A4 and Nrf2 Expression to Detoxify the Toxicity Induced by PQ

To evaluate the detoxification effect of licorice on PQ-induced lung injury, the level of Nrf2 and CYP3A4 expression of lung tissues and CYP3A4 expression of liver tissues was measured by western blotting. Compared with the control group, PQ decreased CYP3A4 and Nrf2 expression remarkably (*P* < 0.01) in the model group. Nrf2 expression was increased after the treatment of licorice extract (60 mg/kg) significantly (*P* < 0.01). It is speculated that the activation of Nrf2 can induce the activation of detoxification pathways. CYP3A4 expression was increased after the treatment of licorice extract (60 mg/kg) significantly (*P* < 0.01), suggesting that licorice may promote the detoxification of paraquat *in vivo* by upregulating CYP3A4, reduce the time of paraquat action *in vivo*, and reduce or detoxify the toxicity of PQ (Figure 5).

### 3.7. Licorice Reduces PQ Accumulation in Mice

**Figure 7:**
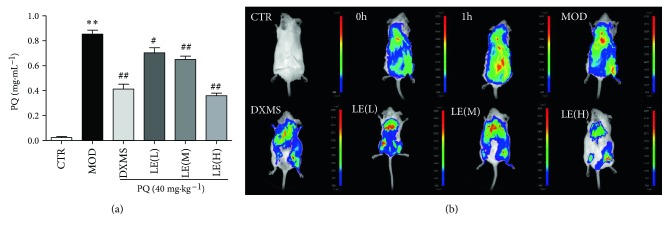
Licorice reduces PQ accumulation in mice. (a) 2MB@CB[8] was used to investigate the concentration of PQ in plasma. After calculating the concentration of PQ in mouse plasma using the method in the literature, it can be found that PQ accumulation in plasma of mice decreased in a dose-dependent manner after treatment of licorice extract. (b) Fluorescence imaging of PQ in living mice. Excitation wavelengths of 580 ± 20 nm were used and the fluorescence emission was detected at 680 ± 30 nm in a Berthold chamber. According to real-time *in vivo* fluorescence imaging of PQ in the living mice, it can be seen that PQ can be readily tracked by using 2MB@CB[8] in living mice. The fluorescence spectra showed that the accumulation of PQ in the licorice extract (60 mg/kg) group was significantly lower than that in the control group. All data are expressed as the mean ± standard error; *n* = 6. CTR: control; MOD: model. Compared with the CTR group, ^∗^ *P* < 0.05, ^∗∗^ *P* < 0.01; compared with the MOD group, ^#^ *P* < 0.05, ^##^ *P* < 0.01.

After calculating the concentration of PQ in mouse plasma using the method in the literature, it can be found that PQ accumulation in plasma of mice decreased in a dose-dependent manner after treatment of licorice extract. This may be related to the upregulated expression of CYP3A4 and Nrf2 underlying the alexipharmic mechanisms of licorice (*P* < 0.01) (Figure 7).

According to real-time *in vivo* fluorescence imaging of PQ in the living mice (Figure 7), it can be seen that PQ can be readily tracked by using 2MB@CB[8] in living mice. The fluorescence spectra showed that the accumulation of PQ in the licorice extract (60 mg/kg) group was significantly lower than that in the control group.

### 3.8. Licorice Improves Liver and Renal Function in PQ-Poisoned Mice

**Figure 8:**
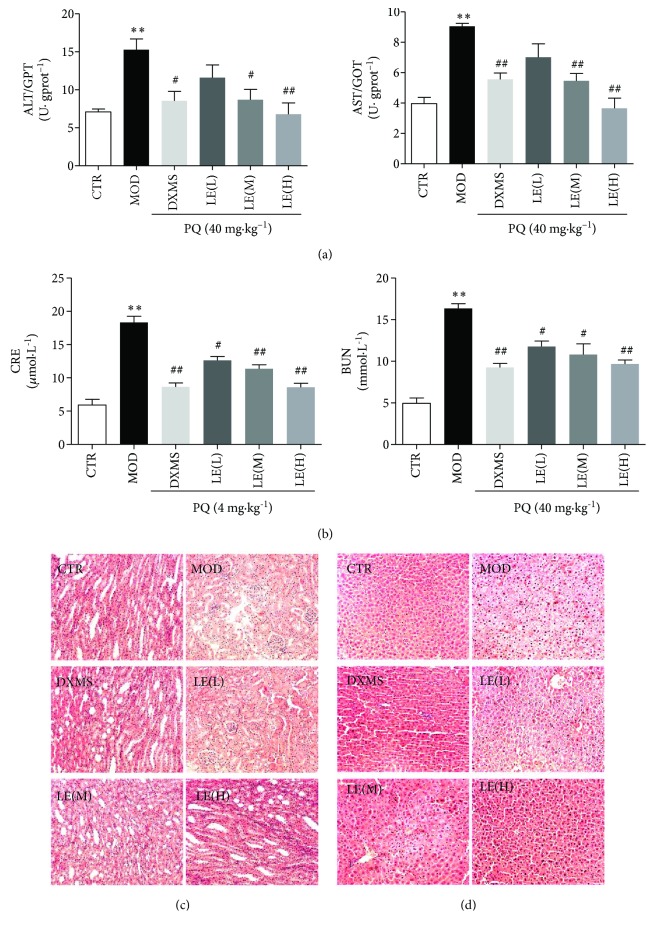
Function and morphology of liver and renal tissues in mice. (a) The levels of alanine aminotransferase (ALT/GPT) and aspartate aminotransferase (AST/GOT) in serum. According to the elevated levels of AST/GOT and ALT/GPT in mice in the model group, the liver function of mice after intake of paraquat was damaged (*P* < 0.01). After treatment with licorice extract, the levels of AST/GOT and ALT/GPT tended to control group and decreased in a dose-dependent manner, especially in licorice extract (60 mg/kg) (*P* < 0.01). (b) The level of renal function was reflected by blood urea nitrogen (BUN) and creatinine (CRE) levels. Data are means ± SEM; *n* = 8. Compared with the CTR group, ^∗^ *P* < 0.05, ^∗∗^ *P* < 0.01; compared with the MOD group, ^#^ *P* < 0.05, ^##^ *P* < 0.01. Compared with the control group, the model group had increased BUN and CRE significantly (*P* < 0.01). Compared with the model group, the licorice extract groups had decreased BUN especially in the licorice extract (60 mg/kg) group (*P* < 0.01). (c, d) H&E staining results of renal and liver tissues. The pathological results of renal and liver tissues showed that no abnormalities were found in normal control; the morphological injury was severe in the paraquat group, which was alleviated in the licorice extract (60 mg/kg) group. Liver and renal sections were analyzed by hematoxylin and eosin (H&E) staining (magnification is ×200).

The levels of aspartate aminotransferase (AST/GOT) and alanine aminotransferase (ALT/GPT) in serum can help people diagnose if the liver is injured or not. When the liver is diseased or damaged, additional AST and ALT are released into the bloodstream, causing levels of the enzyme to rise. Therefore, the amount of AST and ALT in the blood is directly related to the extent of the tissue damage [39]. According to the elevated levels of AST/GOT and ALT/GPT in mice in the model group, the liver function of mice after intake of paraquat was damaged (*P* < 0.01). After treatment with licorice extract, the levels of AST/GOT and ALT/GPT tended to control group and decreased in a dose-dependent manner, especially in licorice extract (60 mg/kg) (*P* < 0.01) (Figure 8(a)). Licorice extract had a protective effect against paraquat-induced liver damage in mice.

The level of renal function was reflected by blood urea nitrogen (BUN) and creatinine (CRE) levels. Compared with the control group, the model group had increased BUN significantly (*P* < 0.01). Compared with the model group, the licorice extract groups had decreased BUN especially in the licorice extract (60 mg/kg) group (*P* < 0.01). A significant difference was observed in serum BUN when comparing the licorice extract (60 mg/kg) group with the model group (Figure 8(b)). Serum CRE increased rapidly after treatment of paraquat (*P* < 0.01). After treatment with licorice extract, CRE level decreased in a dose-dependent manner especially in the licorice extract (60 mg/kg) group (*P* < 0.01). The result displays a protective effect against paraquat-induced renal damage in mice. The mechanism of the protection appears to be the induction of CYP3A4 and Nrf2 expression.

From the H&E staining results of the renal tissue, normal renal structure was observed in the control group. In the mice with paraquat, tissue necrosis and hemorrhage and significant tissue injury were observed also with tubular and glomerular necrosis. Administration of licorice extract (60 mg/kg) improved tissue structure; glomeruli and tubules were better preserved (Figure 8(c)).

Liver sections were analyzed for histopathological changes. Sections from the control group had radially arranged normal hepatocytes with intact central veins and bile ducts. Increased sinusoidal spaces and congestion, damaged central vein and bile ducts, loss of cell boundaries, variable degrees of vacuolization, degenerating nuclei, and apoptotic cells were present in the paraquat group. Licorice extract treatment, especially at a dosage of 60 mg/kg, somewhat preserved the normal liver histology (Figure 8(d)).

### 3.9. Potential Active Target Searching and Clustering Analysis of Network Topology Relationship by MCODE

**Figure 9:**
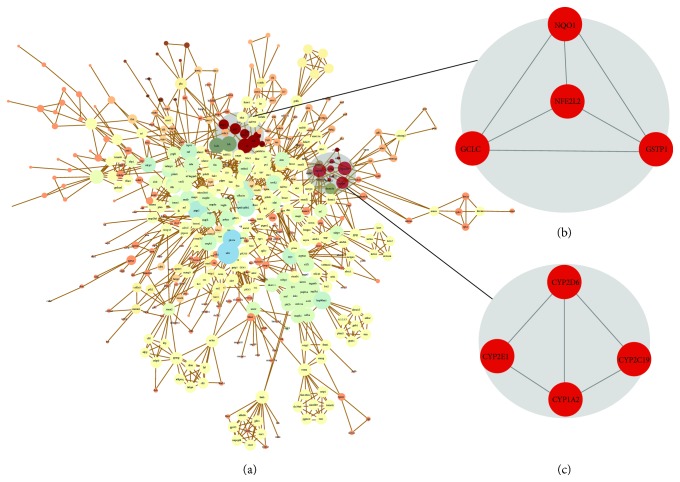
Network construction of protein interaction in licorice ingredients and paraquat. The ingredients of OB ≥ 30% and DL ≥ 0.1 in licorice through TCMSP database (Supplementary Table 1) and PQ were submitted to the Agilent Literature Search software v.3.1.1 (http://www.agilent.com/labs/research/litsearch.html), which is a powerful automatic metasearch tool for querying multiple text-based PubMed and USPTO, for associations among genes of interest and constructing a network. Agilent Literature Search is a registered plugin and can be used in conjunction with Cytoscape 3.2 (accessed on May 1, 2015, http://www.cytoscape.org/) to realize the visualization and analyzation of the network (parameters: Max Engine Matches = 10; Concept Lexicon = Homo sapiens; Interaction Lexicon = limited). **(**a) Agilent Literature Search Network of 104 common genes from licorice and PQ based on Cytoscape; red ellipses indicate downstream genes of Nrf2 and CYPs, whose seed genes are included in our list. (b) NFE2L2 (Nrf2) and (c) CYP1A2 seed nodes, respectively. All edges represent physical interactions. And there is a synergy between Nrf2 and CYPs; among them, GSTP1 is the key target to connect the two groups (Supplementary Figure 6).

There are 104 common targets between licorice and paraquat. The Agilent Literature Search networks of the expressed genes contained 606 nodes and 1739 edges (Figure 9) (Supplementary Table 1). Connections contain direct interaction partners and interconnections. Cytoscape analysis revealed a great number of close interconnections that can be seen in Figure 5. MCODE was used to identify the hub genes or proteins in the networks with a connectivity degree ≥ 3.333 (Supplementary Table 2). Twenty-eight hub genes were selected from the network, which included Apoptosis regulator NFE2L2 (Nrf2) and CYP2B6. There are 36 oxidoreductases that express string and Nrf2 as the core of the region to CYP450 connected to the network and in the center. Prove two important targets Nrf2 and CYP450 in licorice and PQ (Supplementary Figure 6).

### 3.10. Network Screening of Active Ingredients

There were 101 ingredients in licorice with the filter conditions of OB ≥ 30% and DL ≥ 0.1 according to the TCMSP website. Fifty-five ingredients could potentially deplete GSH, and 50 ingredients could react with the CYP3A4 substrate according to the admetSAR website (accessed on May 1, 2015, http://lmmd.ecust.edu.cn/admetsar1). Ingredients have high total score binding to CYP3A4. Ultimately, 28 ingredients could react both ways and these were divided into the following five classes: 6 coumarins, 3 flavonoids, 14 isoflavones, 4 chalcones, and 1 triterpene (Supplementary Table 3). We chose GLL, ILF, LCA, LIA, and 18*β*-GA, the five ingredients in licorice to represent the five classes. GLL was chosen due to the high OB. LCA was chosen due to the high total score in molecular docking. 18*β*-GA has been reported and is responsible for the concentration-dependent induction of CYP3A and Nrf2 [18].

### 3.11. Effect of CYP3A4 in Active Ingredients of Licorice Evaluated by Molecular Docking

**Figure 10:**
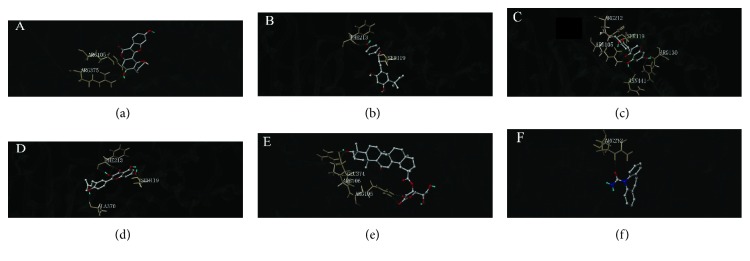
Compounds docked to the catalytic site of CYP3A4. The drug is shown in stick form. The broken lines represent hydrogen bonds. Interacting amino acid residues are labeled (a) GLL, (b) LCA, (c) LIA, (d) ILF, (e) 18*β*-GA, and (f) carbamazepine.

The virtual docking results indicated that LCA exhibited characteristics of strong binding to the catalytic domain of CYP3A4. The total score was determined to be 6.0907. Other ingredients, namely, GLL, LIA, ILF, and 18*β*-GA, were also used as ligands to interact with the catalytic domain of CYP3A4 (Figure 10). The total score of ILF, LIA, GLL, 18*β*-GA, and carbamazepine was determined to be 6.0287, 5.6487, 4.7912, 4.4983, and 4.3030, respectively. LIA and carbamazepine also interact with ARG212. GLL, LIA, and 18*β*-GA interact with the same ligands ARG105. SER119 was interacted with LCA, LIA, and ILF (Figure 10) (Supplementary Table 4).

### 3.12. Effects of the Five Ingredients on A549 and HepG2 Cell Proliferation: A549 and HepG2 Cells Are More Sensitive to PQ with Nrf2 and CYP3A4 Inhibitor

**Figure 11:**
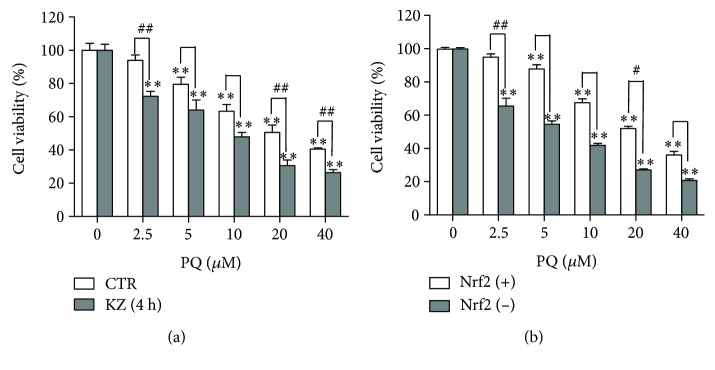
A549 and HepG2 cells more sensitive to PQ with Nrf2 and CYP3A4 inhibitor. (a) HepG2 cell pretreatment with 10 *μ*M ketoconazole for 4 h and exposure to 0-40 *μ*M PQ for 24 h and HepG2 cell exposure to 0-40 *μ*M PQ for 24 h. (b) Cell viability. A549 cells and Nrf2 knockdown A549 cells (5.0 × 10^4^) were incubated with the indicated concentrations of PQ for 24 h. All data are expressed as the mean ± standard error; *n* = 4. CTR: control. ^#^ *P* < 0.05 and ^##^ *P* < 0.01 vs. A549 and HepG2 group; ^∗^ *P* < 0.05 and ^∗∗^ *P* < 0.01 vs. control group.

**Figure 12:**
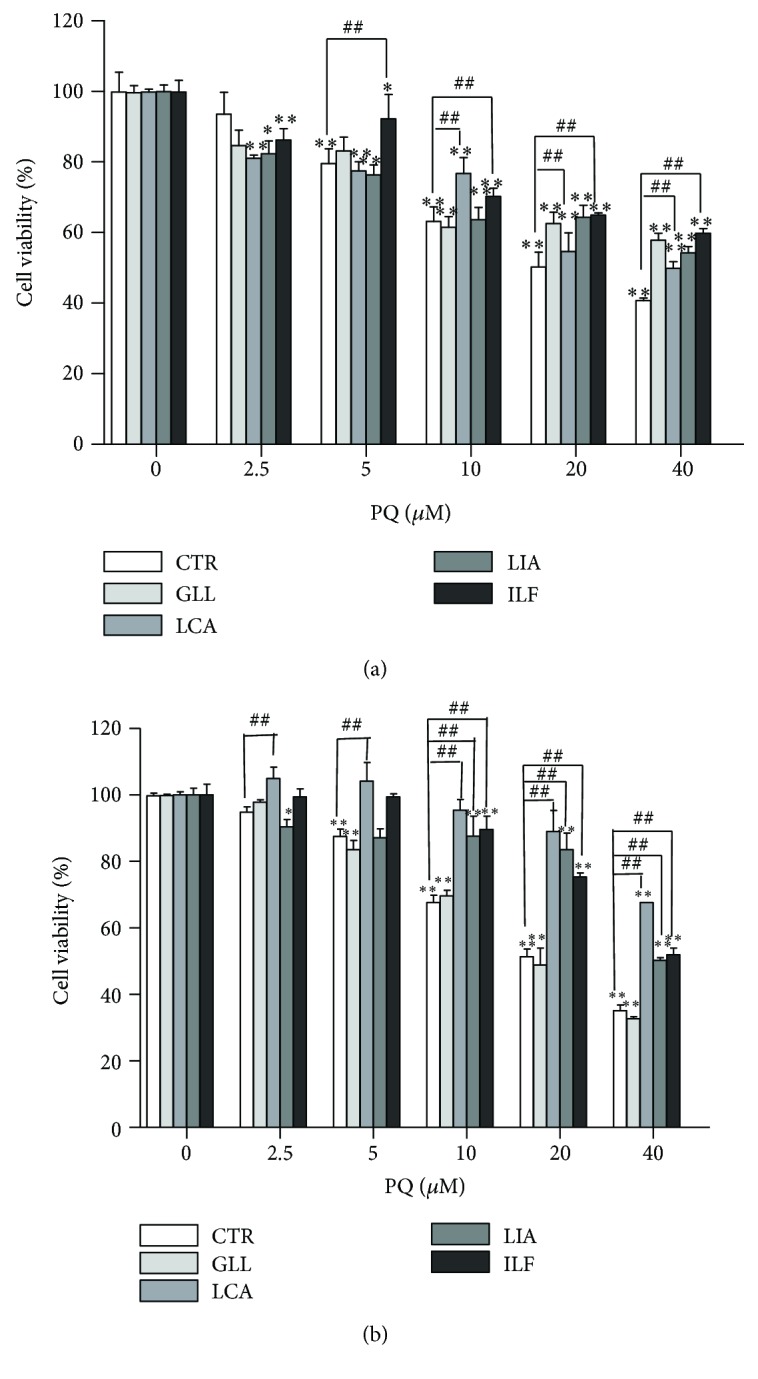
LCA, LIA, and ILF reduced viability when PQ concentration increased. (a) HepG2 cell pretreatment with 10 *μ*M GLL, LCA, LIA, and ILF for 6 h and exposure to 0-40 *μ*M PQ for 24 h. (b) A549 cell pretreatment with 10 *μ*M GLL, LCA, LIA, and ILF for 6.0 h and exposure to 0-40 *μ*M PQ for 24 h. CTR: control. ^#^ *P* < 0.05 and ^##^ *P* < 0.01 vs. pharmacological preconditioning group and the blank group; ^∗^ *P* < 0.05 and ^∗∗^ *P* < 0.01 vs. control group.

**Figure 13:**
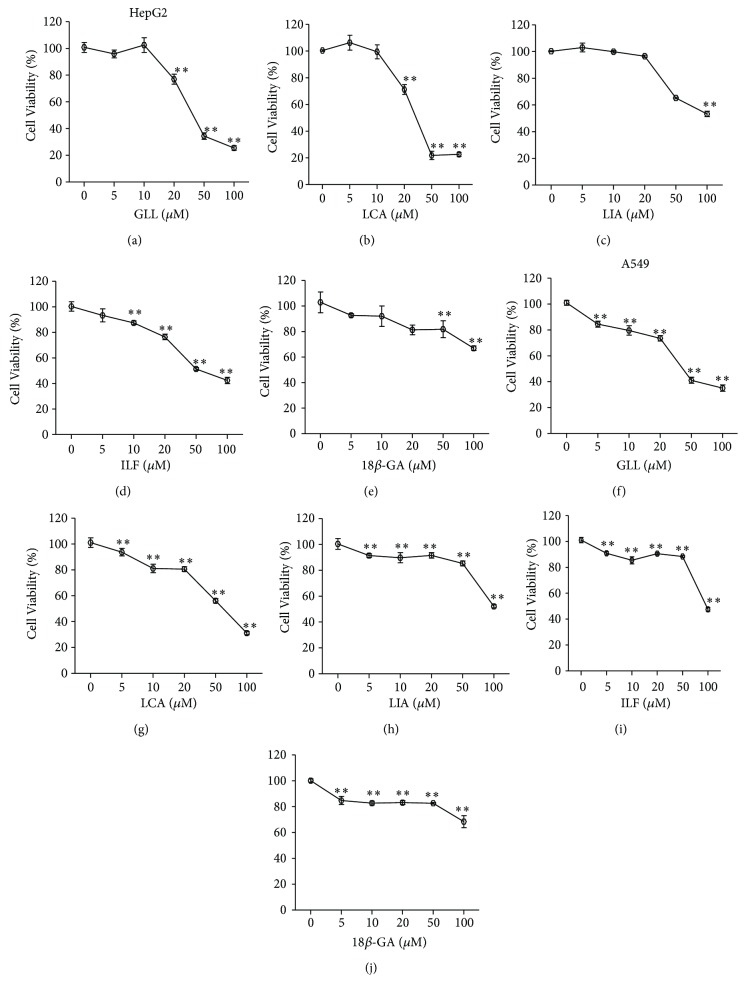
Cell viability. (a) HepG2 treated with GLL. (b) HepG2 treated with LCA. (c) HepG2 treated with LIA. (d) HepG2 treated with ILF. (e) HepG2 treated with 18*β*-GA. (f) A549 treated with GLL. (g) A549 treated with LCA. (h) A549 treated with LIA. (i) A549 treated with ILF. (j) A549 treated with 18*β*-GA. All data are expressed as the mean ± standard error; *n* = 4. CTR: control. ^∗^ *P* < 0.05 and ^∗∗^ *P* < 0.01 vs. control group.

HepG2 cell pretreatment with 10 *μ*M KZ significantly reduced viability compared with HepG2 cells without KZ when PQ concentration increased (Figure 11(a)). KZ reversely verified the activation of the hepatic drug-metabolizing enzyme capable of solution of PQ toxicity. Nrf2 knockdown of A549 cell viability was significantly reduced compared with A549 cells with increased PQ concentration (Figure 11(b)). A549 cell pretreatment with 10 *μ*M LCA, LIA, and ILF significantly reduced viability compared with A549 cell control group when PQ concentration increased and LCA increased the viability more than the other ingredients. HepG2 cell pretreatment with 10 *μ*M LCA and ILF also reduced viability (Figures 12(a) and 12(b)). It verified the activation of the hepatic drug-metabolizing enzyme capable of solution of PQ toxicity. With 5 *μ*M and 10 *μ*M of GLL, ILF, LCA, and LIA and 50 *μ*M and 100 *μ*M of 18*β*-GA added, there was lower cytotoxic activity after 24 h of incubation, whereas higher concentrations of these ingredients decreased the proliferation of HepG2 and A549 cells in a concentration-dependent manner after 24 h of incubation (Figure 13).

### 3.13. Five Ingredients Induced Not Only the Activation of Nrf2, GR, Gpx, and NQO1 mRNA Expression in A549 Cells But Also CYP3A4 mRNA Expression and CYP3A4 Protein in HepG2 Cells

**Figure 14:**
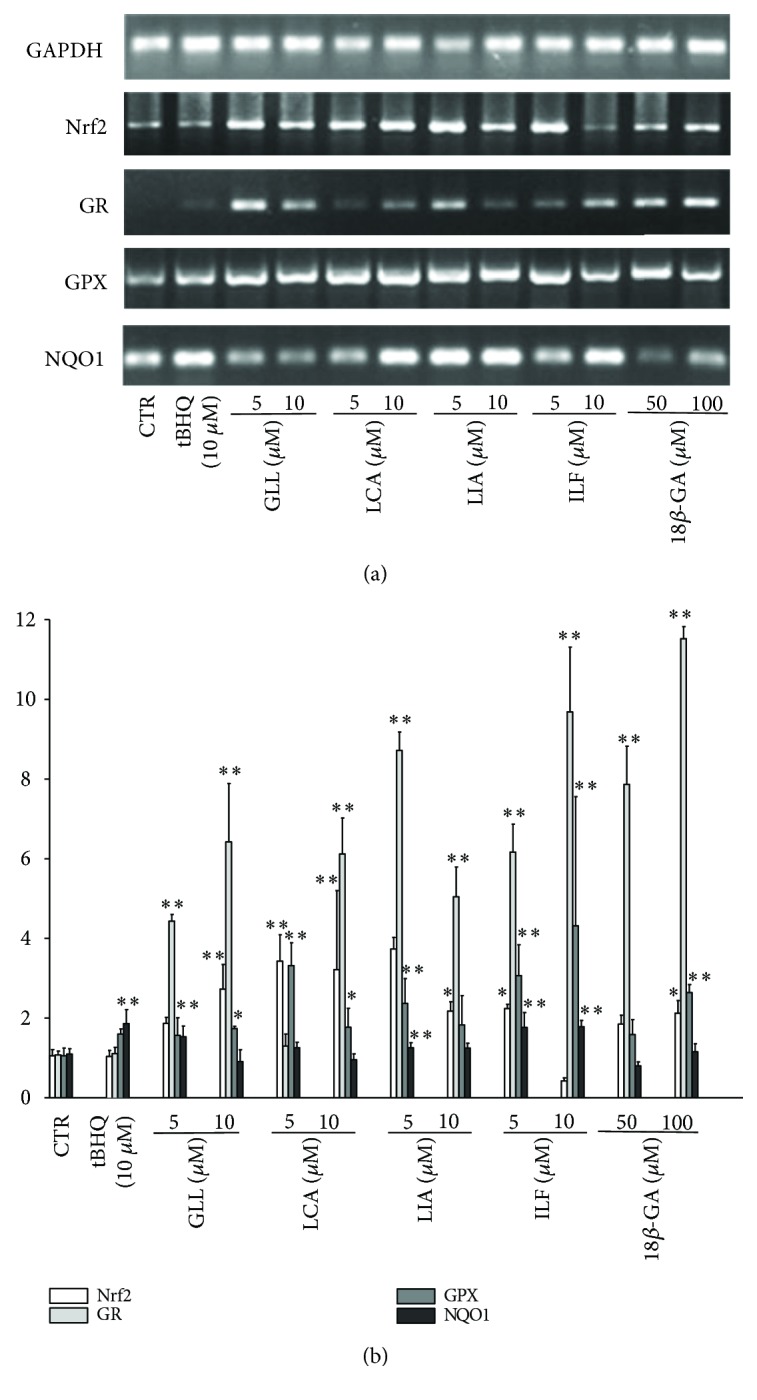
Effects of the five compounds on Nrf2, GR, GPX, and NQO1 mRNA expression in A549 cells. (a) A549 cells were incubated with 5 or 10 *μ*M GLL, ILF, LCA, and LIA and 50 or 100 *μ*M 18*β*-GA. Those expression levels were monitored by semiquantitative RT-PCR assay. (b) The expression levels were monitored by quantitative real-time RT-PCR. Data points and error bars represent the mean ± S.E.M. of at least three experiments (*n* = 3) for each concentration tested; (^∗^ *P* < 0.05).

RT-PCR was performed after 8 h of incubation with the five selected ingredients and the levels of mRNA expression were determined. Compared with the control group, Nrf2, GR, GPX, and NQO1 mRNA expressions increased after 8 h exposure to 5 *μ*M and 10 *μ*M GLL, LCA, LIA, and ILF and 50 *μ*M and 100 *μ*M 18*β*-GA with lower cytotoxic activity. Treatment with GLL and 18*β*-GA resulted in enhanced expression of Nrf2 in a dose-dependent manner. 5 *μ*M LIA played a critical role in activating Nrf2. Treatment with GLL, LCA, ILF, and 18*β*-GA resulted in enhanced expression of GR in a dose-dependent manner. 5 *μ*M ILF played a critical role in activating GR (Figure 14). We found that as the concentration of the five ingredients increased, there was a rapid increase in Nrf2, GR, GPX, and NQO1 expression levels (Supplementary Table 5).

**Figure 15:**
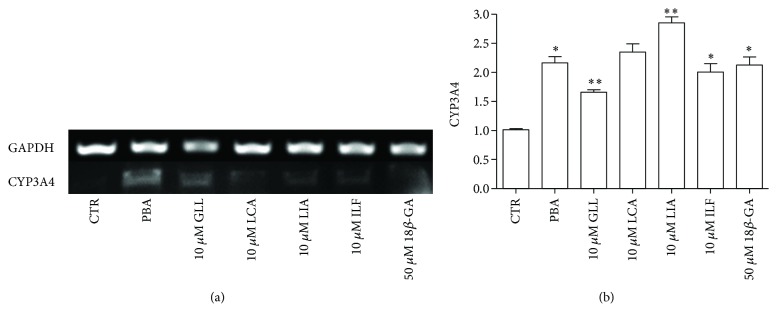
Effects of the five compounds on CYP3A4 mRNA expression in HepG2 cells. (a) HepG2 cells were incubated with 10 *μ*M GLL, LCA, LIA, and ILF and 50 *μ*M 18*β*-GA. Those expression levels were monitored by semiquantitative RT-PCR assay. (b) The expression levels were monitored by quantitative real-time RT-PCR. Data points and error bars represent the mean ± S.E.M. of at least three experiments (*n* = 3) for each concentration tested; (^∗^ *P* < 0.05).

**Figure 16:**
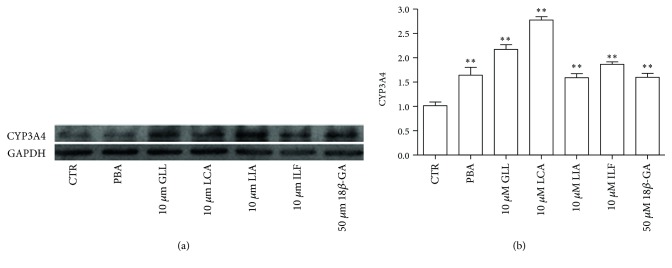
Effects of the five compounds on CYP3A4 expression in HepG2 cells. (a) HepG2 cells were incubated with 10 *μ*M GLL, LCA, LIA, and ILF and 50 *μ*M 18*β*-GA for 24 h. CYP3A4 protein expression was measured by western blotting. (b) Data points and error bars represent the mean ± S.E.M. of at least three experiments (*n* = 3) for each concentration tested; (^∗^ *P* < 0.05).

CYP3A4 was significantly activated by 10 *μ*M GLL, LCA, LIA, and ILF and 50 *μ*M 18*β*-GA, as detected by RT-PCR after 24 h of incubation with the five ingredients. In particular, LIA played a critical role in activating CYP3A4 (Figure 15). Western blot analysis showed that the protein level of CYP3A4 expression was significantly increased compared to that of the control cells following 24 h of exposure to 10 *μ*M GLL, ILF, LCA, and LIA and that 10 *μ*M LCA increased the protein levels of CYP3A4 more than the other ingredients (Figure 16). The total score of LCA is also more than the other ingredients in molecular docking. In summary, our results indicated that GLL, LCA, LIA, and ILF activation of the Nrf2 signaling pathway and its downstream genes, in addition to the activation of the CYP3A4, could be one of the detoxification mechanisms in licorice.

### 3.14. Five Ingredients Downregulated GCLM and NQO1 mRNA Expression in Nrf2-Silenced A549 Cells

**Figure 17:**
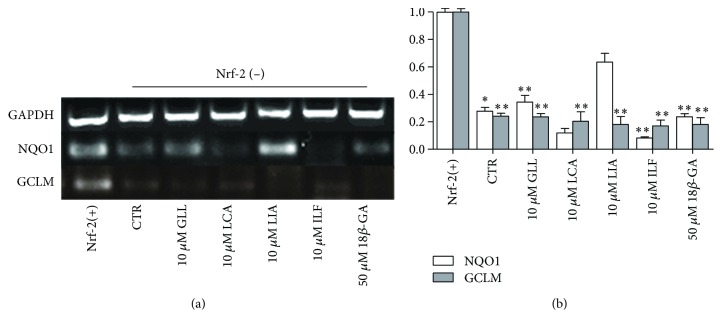
Effects of the five compounds on GCLM and NQO1 mRNA expression in A549 cells. (a) A549 cells were incubated with 10 *μ*M GLL, LCA, LIA, and ILF and 50 *μ*M 18*β*-GA. Those expression levels were monitored by semiquantitative RT-PCR assay. (b) The expression levels were monitored by quantitative real-time RT-PCR. Data points and error bars represent the mean ± S.E.M. of at least three experiments (*n* = 3) for each concentration tested; (^∗^ *P* < 0.05).

In order to understand the alexipharmic role of CYP3A4 and Nrf2, we used Nrf2-silenced A549 cell line for further study. We then transfected cells with Nrf2 siRNA. Compared with the control and shRNA-NC, the Nrf2 gene-silenced two A549 cell clones displayed a declining trend in the expressions of the glutathione pathway genes; GCLM and NQO1 were downregulated. RT-PCR was performed after 8 h of incubation with the five ingredients and mRNA expression levels were determined. Compared with the control group, GCLM mRNA expression did not increase following 8 h of exposure to 10 *μ*M GLL, LCA, LIA, and ILF and 50 *μ*M 18*β*-GA. NQO1 mRNA expression levels did not increase compared to those of the control vector transfected cells following 8 h of exposure to 10 *μ*M LCA and LIA and 50 *μ*M 18*β*-GA but did moderately increase with exposure to LIA (Figure 17).

## 4. Discussion

PQ is highly toxic to humans and induces extensive pulmonary injury, including pulmonary edema, hypoxemia, respiratory failure, and pulmonary fibrosis [40]. Generally, acute lung injury is diffused heterogeneous lung injury characterized by hypoxemia, noncardiogenic pulmonary edema, low lung compliance, and widespread capillary leakage [41]. At present, the mechanism of PQ-induced ALI is still not conclusively known. Most scholars believe that PQ induces its toxic effects mainly by oxidative stress which has an important function in this process. In oxidative stress process, PQ consumes nicotinamide adenine dinucleotide phosphate and cytochrome P450 reductase, causing oxidative stress reaction. It told us that antioxidation and detoxification are important in the treatment of paraquat detoxification.

As a famous herbal alexipharmacon, licorice has demonstrated remarkable polypharmcological applications, such as anticancer, anti-inflammatory, and hepatoprotective effects [18]. Recent successful efforts were made on antidotes for various poisonings, including but not limited to, food, herbs, herbicides, pesticides, drugs, and heavy metals [18]. Gene functional classification illustrates correlated expression patterns and because of the importance of networks in system biology, quantitative tools have been developed in recent years for analyzing these networks [28]. In the present study, we use oxidative stress biomarkers and detoxifying enzymes to measure the protective effect of licorice on PQ-induced acute lung injury *in vivo*. To study the mechanism of detoxification and antioxidation, we use a system pharmacology approach to determine the active components in licorice [42]. As depicted in the results, *in vivo* studies indicated that licorice alleviate pulmonary edema and fibrosis, decrease MDA contents and increase SOD activity in PQ-induced ALI mice, protect the morphologic appearance of lung tissues, induce CYA3A4 and Nrf2 expression to active detoxification pathways, reduce the accumulation of PQ *in vivo*, protect or improve the liver and renal function of mice, and increase the survival rate. With the positive effect *in vivo*, we explore the possible mechanisms of action and obtained four unreported candidate compounds *in silico*. GLL (OB = 90.78%) is a natural compound extracted from *Glycyrrhiza uralensis*. IFL (OB = 45.17%) was identified and considered to be an active inhibitor against angiogenesis and a promising compound as a potential cancer chemopreventive agent [43]. LIA (OB = 41.61%) was identified for its antibacterial and gastroprotective properties [44]. At low concentrations, it inhibits MRP-mediated efflux to control MRP-like activity in human erythrocytes. LCA (OB = 40.79%) has been shown to exhibit multiple pharmacological properties [45].

Analyzing the network properties of gene expression data might reveal the organizational pattern of gene expression in licorice, which might, in turn, help to identify the mechanisms of molecular detoxification. Drug-metabolizing enzymes such as CYP450, which help bioactivate toxic substances, and phase II enzyme GST, which can reduce the toxicity of electrophilic compounds formed by phase I enzymes [46]. In a series of redox reactions, PQ consumes nicotinamide adenine dinucleotide phosphate and cytochrome P450 reductase, cause oxidative stress reaction. Hence, the actual effects of the accumulation of toxic metabolic products of drugs and the detoxification process depend on the balance between phase I and phase II enzymes.

Nrf2, a master regulator of the detoxifying response, plays a critical role in the expression of many cytoprotective enzymes, including NQO1, HO-1, and glutathione S-transferase (GST). As represented in Figure 9, there are many proteins with the most degrees as hubs, such as AFF4 (AF4/FMR2 family, member 4), Nrf2, and CYP2B6. We selected all oxidoreductase to analyze the bond that linked them. In Supplementary Figure 6, GST is the more important protein as a link between CYP450 and Nrf2. According to the literature, GST may account for the variation in host response to oxidative stress, a key component of airway inflammation [47]. Previous studies have shown that Nrf2 protects against endotoxin-induced hepatic injury through transcriptional upregulation of downstream genes, such as GCL, GR, GPX, and NQO1 [48], which suggest that the protective part of Nrf2 is attributed partly to its involvement in coordinated induction of phase II drug-metabolizing enzymes. Studies have shown that overexpression of GR is capable of increasing stress tolerance. Glutathione peroxidase-1 (GPx-1) is an intracellular antioxidant enzyme that enzymatically reduces hydrogen peroxide to water to limit its harmful effects. NQO1, a phase II metabolic enzyme, is known as a target for bioreductive activation of certain anticancer drugs and antioxidants and as a means of xenobiotic detoxification. GCL, the enzyme that catalyzes the rate-limiting step in the biosynthesis of GSH, can combine with toxic components, leading to the excretion of those toxins [21].

The study has shown that drug metabolism and efflux transporters are considered to play important roles in regulating the body. Concurrently, the subnetwork with the seed gene, Nrf2, is a basic leucine zipper transcription factor and is necessary against oxidative stress and regulates the expressions of a variety of drug-metabolizing enzymes [21, 49]. It has been found that Nrf2, an important transcription factor of oxidative stress, is known to be essential for the antioxidant responsive element-mediated induction of oxidative stress enzyme genes and plays an important role in the development of PQ-induced ALI. Many studies showed their results by inhibiting oxidative stress to slow down the process of ALI [13]. Studies have revealed that Nrf2 knockout mice were more susceptible to butylated hydroxytoluene-induced lung injury and acetaminophen-induced hepatotoxicity at high doses [50]. It was identified that the RNAi-mediated reduction of Nrf2 expression in lung cancer cells induces the generation of ROS, decreases the level of reduced glutathione, and results in an increase in the A549 cell proliferation inhibition rate. In corroboration with GR, GPX and GST gene expression as well as the total GSH levels was significantly reduced in Nrf2 shRNAi A549 cells and in comparison with the shRNA-NC control cells. Nrf2 expression downregulation profoundly decreased the expression of key antioxidative enzyme genes, glutathione pathway genes with a constructive activation of Nrf2 function. Thus, the licorice-induced expression of Nrf2 can enhance detoxification [30]. As shown in the present study, we successfully disclosed that GLL, LCA, ILF, and LIA significantly induce GR, GPX, and NQO1 mRNA expression (Figure 14), and we first indicated that GLL, ILF, LCA, and LIA induce CYP3A4 mRNA expression (Figure 15) and have subsequent effects on CYP3A4 protein levels (Figure 16). On the basis of total score in molecular docking, LCA was found to be a better CYP3A4 agonist than the other ingredients, which is consistent with our results. However, it is noteworthy to mention that all the other ingredients also showed considerable affinities toward CYP3A4. Hence, structures of these ingredients could be used for designing antidotes against toxic medications. The virtual docking results indicated that the five ingredients exhibited characteristics of strong binding to the catalytic domain of CYP3A4. The five compounds induced CYP3A4 mRNA and protein levels further indicating that the five compounds are CYP3A4 agonists (Supplementary Table 4). It verified the activation of hepatic drug-metabolizing enzyme capable of solution of PQ toxicity through GLL, LCA, LIA, and ILF.

We hope low-concentration compounds of licorice as an emerging paradigm emphasizes molecularly targeted approaches for PQ-induced lung injury therapy. *In vivo* study showed that licorice increase the level of CYP3A4 and Nrf2, alleviate pulmonary edema, injury, and fibrosis, reduce the accumulation of PQ *in vivo*, protect or improve the liver and renal function of mice, and ultimately improve the survival rate of mice. GLL, ILF, LCA, 18*β*-GA, and LIA isolated from licorice significantly induce Nrf2 and its downstream detoxification genes GR, GPX, and NQO1. We can infer that the other active components in licorice may also have the potential activity of detoxification on paraquat poisoning through *in vivo* experiments. And the content of four new compounds of licorice after *in silico* screening is worth exploring. Perhaps the effect of drug combination between detoxification molecules will be better. Notably, these data might represent, at least in part, a new mechanism by which the traditional use of licorice can alleviate the side effects of toxic TCMs.

## 5. Conclusion

The current study constitutes the antioxidation and detoxification effects of licorice extract on PQ-induced acute lung injury, alleviating pulmonary edema and fibrosis, reducing the accumulation of PQ *in vivo*, protecting or improving liver and renal function of mice, and finally, improving the survival rate of mice. For the first report, GLL, ILF, LCA, and LIA increased the level of CYP3A4 and Nrf2 as a mechanism for detoxification. Moreover, GLL was found to be a better CYP3A4 agonist than the other ingredients.

## Data Availability

All data generated or analyzed during this study are included in this published article and its supplementary information files.

## Abbreviations

A549: The human type II alveolar adenocarcinomic basal epithelial cells
ALI: Acute lung injury
ADMET: Absorption, Distribution, Metabolism, Excretion, and Toxicity
AFF4: AF4/FMR2 family, member 4
AKT-1: RAC-alpha serine/threonine-protein kinase
BAX: Apoptosis regulator BAX
CYP450: Cytochrome P450
CYP3A4: Cytochrome 3A4
DMEM/F12: Dulbecco's Modified Eagle's Medium Nutrient Mixture F-12
DL: Drug-likeness
GAPDH: Glyceraldehyde-3-phosphate dehydrogenase
GCLM: Glutamate cysteine ligase modifier
GLL: Glycyrol
GPX: Glutathione peroxidase
GR: Glutathione reductase
GSH: Glutathione
HepG2: Human hepatocellular liver carcinoma cell
HP: Hemoperfusion
ILF: Isolicoflavonol
KZ: Ketoconazole
LE: Licorice extract
LCA: Licochalcone A
LIA: Licoisoflavone A
MCODE: Molecular Complex Detection
MDA: Malondialdehyde
MEM: Minimum Essential Medium
MTT: 3-(4.5-Dimethylthiazol-2-yl)-2.5-diphenyl tetrazolium bromide
Nrf2, NFE2L2: Nuclear factor erythroid 2-related factor 2
NQO1: NAD (P)H:quinoneoxidoreductase
OB: Oral bioavailability
PQ: Paraquat
QSAR: Quantitative structure-activity relationship
RT-PCR: Real-Time PCR
SFRP4: Secreted frizzled-related protein 4
SOD: Superoxide Dismutase
TCMSP: Traditional Chinese Medicine Systems Pharmacology Database and Analysis Platform
TCM: Traditional Chinese medicine
18*β*-GA: 18Beta-glycyrrhetinic acid.

## Supplementary Materials

Supplementary Materials Supplementary Table 1: the ingredients of *OB* ≥ 30% and *DL* ≥ 0.1 in licorice and nodes in Agilent Literature Search. Supplementary Table 2: MCODE scoring for PPI network. Molecular docking was used as a tool to screen for ingredients having strong energy binding to CYP3A4, which is a member of the cytochrome P450 family of oxidizing enzymes. MCODE was used to identify the hub genes or proteins in the networks with a connectivity degree ≥ 3.333. Supplementary Table 3: active compounds which could potentially deplete GSH and have high total score binding to CYP3A4 according to the admetSAR website. Supplementary Table 4: molecular docking score for ingredients that have strong energy binding to CYP3A4. Supplementary Table 5: Real-Time PCR primer sequences. Supplementary Figure 6: there are 36 oxidoreductases that express string and Nrf-2 as the core of the region to CYP450 connected to the network and in the center. Prove two important targets Nrf-2 and CYP450 in licorice and PQ. Supplementary Figure 7: the effect of licorice extract on levels of MDA and SOD. Effects of treatment with licorice extract on the levels of MDA (A) and SOD activity (B) of the lung tissue. Treatment with licorice extract reduced the level of MDA and increased the activity in the lung tissues of PQ-induced lung injury. But in LE (80 mg/kg), MDA and SOD levels were increased compared with LE (60 mg/kg). Data are means ± SEM; *n* = 8. ^∗^ *P* < 0.05 and ^∗∗^ *P* < 0.05 compared with the CTR group, ^#^ *P* < 0.05 and ^#^^#^ *P* < 0.01 compared with the MOD group. Supplementary Figure 8: DXMS docked to the catalytic site of CYP3A4.
