# Supplementary material for: The Alexipharmic Mechanisms of Five Licorice Ingredients Involved in CYP450 and Nrf2 Pathways in Paraquat-Induced Mice Acute Lung Injury
Source: Oxid Med Cell Longev. 2019 Apr 28;2019:7283104. doi: 10.1155/2019/7283104 (PMC6512064; doi:10.1155/2019/7283104)
Supplement: Supplementary Materials — Supplementary Table 1: the ingredients of OB ≥ 30% and DL ≥ 0.1 in licorice and nodes in Agilent Literature Search. Supplementary Table 2: MCODE scoring for PPI network. Molecular docking was used as a tool to screen for ingredients having strong energy binding to CYP3A4, which is a member of the cytochrome P450 family of oxidizing enzymes. MCODE was used to identify the hub genes or proteins in the networks with a connectivity degree ≥ 3.333. Supplementary Table 3: active compounds which could potentially deplete GSH and have high total score binding to CYP3A4 according to the admetSAR website. Supplementary Table 4: molecular docking score for ingredients that have strong energy binding to CYP3A4. Supplementary Table 5: Real-Time PCR primer sequences. Supplementary Figure 6: there are 36 oxidoreductases that express string and Nrf-2 as the core of the region to CYP450 connected to the network and in the center. Prove two important targets Nrf-2 and CYP450 in licorice and PQ. Supplementary Figure 7: the effect of licorice extract on levels of MDA and SOD. Effects of treatment with licorice extract on the levels of MDA (A) and SOD activity (B) of the lung tissue. Treatment with licorice extract reduced the level of MDA and increased the activity in the lung tissues of PQ-induced lung injury. But in LE (80 mg/kg), MDA and SOD levels were increased compared with LE (60 mg/kg). Data are means ± SEM; n = 8. ∗ P < 0.05 and ∗∗ P < 0.05 compared with the CTR group, # P < 0.05 and # # P < 0.01 compared with the MOD group. Supplementary Figure 8: DXMS docked to the catalytic site of CYP3A4. [file 7283104.f1.zip › Supplementary Data/Supplementary Table 1.pdf]

Supplementary Table 1. The ingredients of OB≥30% and DL≥0.1 in licorice and have nodes in Agilent Literature Search.

Mol ID	Molecule Name	Nodes
MOL003656	Lupiwighteone	3
MOL000417	Calycosin	60
MOL000422	Kaempferol	202
MOL004328	Naringenin	233
MOL004806	Euchrenone	2
MOL004841	Licochalcone B	10
MOL004879	Glycyrin	4
MOL004908	Glabridin	85
MOL000098	Quercetin	222
MOL000497	LCA	110
MOL001789	Isoliquiritigenin	169
MOL002311	GLL	13
MOL002565	Medicarpin	33
MOL002844	Pinocembrin	67
MOL000354	Isorhamnetin	127
MOL000359	Sitosterol	96
MOL000392	Formononetin	111
MOL004985	Icos-5-enoic acid	291
MOL005016	Odoratin	2
MOL004957	Isoformononetin	2
MOL001792	Liquiritigenin	65
