# Supplementary material for: The Alexipharmic Mechanisms of Five Licorice Ingredients Involved in CYP450 and Nrf2 Pathways in Paraquat-Induced Mice Acute Lung Injury
Source: Oxid Med Cell Longev. 2019 Apr 28;2019:7283104. doi: 10.1155/2019/7283104 (PMC6512064; doi:10.1155/2019/7283104)
Supplement: Supplementary Materials — Supplementary Table 1: the ingredients of OB ≥ 30% and DL ≥ 0.1 in licorice and nodes in Agilent Literature Search. Supplementary Table 2: MCODE scoring for PPI network. Molecular docking was used as a tool to screen for ingredients having strong energy binding to CYP3A4, which is a member of the cytochrome P450 family of oxidizing enzymes. MCODE was used to identify the hub genes or proteins in the networks with a connectivity degree ≥ 3.333. Supplementary Table 3: active compounds which could potentially deplete GSH and have high total score binding to CYP3A4 according to the admetSAR website. Supplementary Table 4: molecular docking score for ingredients that have strong energy binding to CYP3A4. Supplementary Table 5: Real-Time PCR primer sequences. Supplementary Figure 6: there are 36 oxidoreductases that express string and Nrf-2 as the core of the region to CYP450 connected to the network and in the center. Prove two important targets Nrf-2 and CYP450 in licorice and PQ. Supplementary Figure 7: the effect of licorice extract on levels of MDA and SOD. Effects of treatment with licorice extract on the levels of MDA (A) and SOD activity (B) of the lung tissue. Treatment with licorice extract reduced the level of MDA and increased the activity in the lung tissues of PQ-induced lung injury. But in LE (80 mg/kg), MDA and SOD levels were increased compared with LE (60 mg/kg). Data are means ± SEM; n = 8. ∗ P < 0.05 and ∗∗ P < 0.05 compared with the CTR group, # P < 0.05 and # # P < 0.01 compared with the MOD group. Supplementary Figure 8: DXMS docked to the catalytic site of CYP3A4. [file 7283104.f1.zip › Supplementary Data/Supplementary Table 3.pdf]

Supplementary Table 3. Active compounds

Mol ID	Molecule Name	OB (%)	DL	Classification	Structure
MOL002311	GLL	90.78	0.67	Coumarin	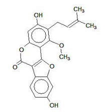
MOL004904	Llicopyranocoumarin	80.36	0.65	Coumarin	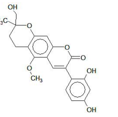
MOL004863	3-(3,4-dihydroxyphenyl)-5,7-dihydroxy-8-(3-methylbut-2-enyl) Chromone	66.37	0.41	Isoflavones	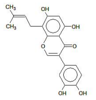
MOL004855	Licoricone	63.58	0.47	Isoflavones	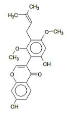
MOL005000	Gancaonin G	60.44	0.39	Isoflavones	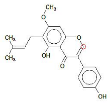
MOL004824	(2S)-6-(2,4-dihydroxyphenyl)-2-(2-hydroxypropan-2-yl)-4-methoxy-2,3-dihydrofuro[3,2-g]chromen-7-one	60.25	0.63	Coumarin	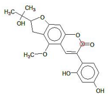
MOL004849	3-(2,4-dihydroxyphenyl)-8-(1,1-dimethylprop-2-enyl)-7-hydroxy-5-methoxy-coumarin	59.62	0.43	Coumarin	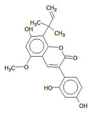
MOL004879	Glycyrin	52.61	0.47	Coumarin	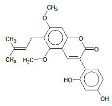
MOL004912	Glabrone	52.51	0.5	Isoflavones	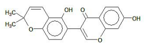
MOL003656	Lupiwighteone	51.64	0.37	Isoflavones	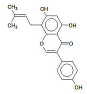
MOL004856	Gancaonin A	51.08	0.4	Isoflavones	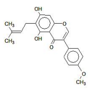
MOL005001	Gancaonin H	50.1	0.78	Isoflavones	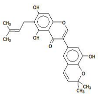

MOL004848	Licochalcone G	49.25	0.32	Chalcones	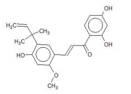
MOL004857	Gancaonin B	48.79	0.45	Isoflavones	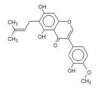
MOL004827	Semilicoisoflavone B	48.78	0.55	Isoflavones	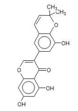
MOL004898	(E)-3-[3,4-dihydroxy-5-(3-methylbut-2-enyl)phenyl]-1-(2,4-dihydroxyphenyl)prop-2-en-1-one	46.27	0.31	Chalcones	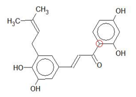
MOL004949	ILF	45.17	0.42	Flavonoids	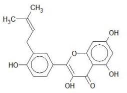
MOL004828	Glepidotin A	44.72	0.35	Flavonoids	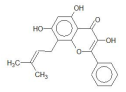
MOL004948	Isoglycyrol	44.7	0.84	Coumarin	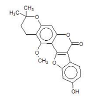
MOL004866	2-(3,4-dihydroxyphenyl)-5,7-dihydroxy-6-(3-methylbut-2-enyl)chromone	44.15	0.41	Flavonoids	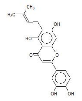
MOL004915	Eurycarpin A	43.28	0.37	Isoflavones	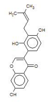
MOL004883	LIA	41.61	0.42	Isoflavones	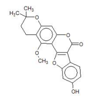
MOL000497	LCA	40.79	0.29	Chalcones	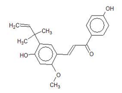
MOL004815	Kanzonol B	39.62	0.35	Chalcones	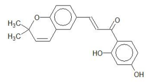
MOL004884	Licoisoflavone B	38.93	0.55	Isoflavones	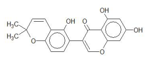
MOL004917	Glycyroside	37.25	0.79	Isoflavones	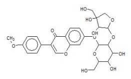

MOL004905	3,22-Dihydroxy-11-oxo-delta(12)-oleanene-27-alpha-methoxycarbonyl-29-oic acid	34.32	0.55	Triterpenes	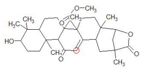
MOL004864	5,7-dihydroxy-3-(4-methoxyphenyl)-8-(3-methylbut-2-enyl)chromone	30.49	0.41	Isoflavones	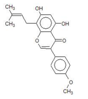

The ingredients of OB≥30% and DL≥0.1 in licorice.Bioavailability play a key role in the drug development period for both new drug products and their generic equivalents. High OB is usually a key indicator of bioactive molecules in juding. (chen et al, 2001). DL is widely used to assess the possibility of compounds becoming drugs. Screening high DL can eliminate the interference of non-pharmaceutical compounds and save the cost and time of drug research and development. (Walters and murcko, 2002). So we rank the OB and DL values from high to low, and the choice of compounds with high oral bioavailability and drug-like properties are very helpful to our drug development and research.
